# Using Smartphones When Eating Increases Caloric Intake in Young People: An Overview of the Literature

**DOI:** 10.3389/fpsyg.2020.587886

**Published:** 2020-12-03

**Authors:** Marco La Marra, Giorgio Caviglia, Raffaella Perrella

**Affiliations:** ^1^Department of Experimental Medicine, University of Campania Luigi Vanvitelli, Naples, Italy; ^2^Department of Psychology, University of Campania Luigi Vanvitelli, Caserta, Italy

**Keywords:** social facilitation, distraction, obesity, food intake, smartphone

## Abstract

Recent literature highlights that the use of smartphones during meals increases the number of calories ingested in young people. Although the distraction interferes with physiological signals of hunger and satiety, a social facilitation effect has also been suggested. Cognition is a pivotal component in regulating food intake, and activities requiring high perceptual demands should be discouraged during meals.

## Introduction

In recent decades, the use of smartphones has gradually increased worldwide. Currently, more than three billion people in the world own a smartphone with an expected increase in the next few years of several hundred million (O’Dea, 2020).

The mobile device has become an essential tool in everyday life, especially among young people, where one in four teenagers claims to use their smartphone constantly (Kabali et al., 2015; Lenhart, 2015).

In the United States, cell phone access in young people has increased from 40% in 2004 to 75% in 2013 (Rideout et al., 2010, 2013), and 53% report having a smartphone from the age of 11, with an increase of more than 80% to 14 years (Anderson and Jiang, 2018; Rideout and Robb, 2019). European figures also record a progressive rise, where 64% of young people have a smartphone at 15–16 years, 55% at 13–14, 40% at 11–12, and 20% at 9–10 years (Mascheroni and Cuman, 2014). Social media applications, in particular, are the most used, with 38% of young people claiming their use several times per hour and 16% interacting with them continuously (Rideout and Robb, 2018).

Linearly related to owning a mobile device, teenagers report a growing increase in media multitasking activities during their ordinary daily activities: in the bathroom, in bed, on the street, and especially during mealtimes (Webby Awards, 2015). This trend does not seem to slow down and could increase further.

Although technological development has led to some improvements across society (such as fast communication and content transmission facilities), certain negative aspects have also been highlighted. These include social isolation, addictive use behavior (Takao et al., 2009) and, more recently, interference with eating behavior, and the amount of calories ingested has been suggested.

Overall, current evidence reveals that the use of digital technologies referred to as “screen time” (devices using a screen) represents an obesity risk factor, especially for young people.

Investigations previously conducted using media devices, such as television and video games, showed a consistent correlation between time spent watching television, body mass index (BMI), and adiposity level (Coon and Tucker, 2002; Janz et al., 2002; Staiano et al., 2013). It was pointed out that people increased their food intake when they were allowed to eat snacks while playing video games or watching television (Temple et al., 2007; Chaput et al., 2011).

More recently, some preliminary studies extend a similar effect to smartphone use (Kenney and Gortmaker, 2017). However, unlike studies conducted with other multimedia devices, research investigating the relationship between food intake and smartphone use is at an early stage. Nevertheless, the American Academy of Pediatrics (2016) expands the effects of television and video games to mobile phones. This is reasonable in the discussion on weight management, since smartphone use—like other forms of screen time device—is also a sedentary activity (Lanningham-Foster et al., 2006). Furthermore, the compact size and one-handed operation make the smartphone the most popular multimedia device currently used during meals.

It has been highlighted that media multitasking via smartphone provides a distracting effect for tasks including reading an article and crossing the street (Stavrinos et al., 2009; Chen and Yan, 2016). Drawing on this observations, in our review, we will analyze the first experimental evidence that smartphone use, similar to other technological devices (Bellisle et al., 2004; Brunstrom and Mitchell, 2006; Hetherington et al., 2006; Robinson et al., 2013), promotes food intake by distracting users from eating behavior.

## Smartphone Distraction and Eating Behavior

Research identifying distraction as something that can promote food intake has been ongoing for quite a few years now. Eating in competition with other tasks has been shown to increase food intake, especially when the tasks are cognitively demanding (Hetherington et al., 2006). For instance, ambient music and the social context in which people consume their meals affect the number of calories ingested (Van der Bilt, 2011; Chapman et al., 2014; Higgs, 2015; Marsh et al., 2015). In a similar vein, experimental evidence indicates that several factors can distract from eating including listening to a story (Bellisle and Dalix, 2001; Long et al., 2011), background music (Stroebele and de Castro, 2006), playing a computer game (Oldham-Cooper et al., 2011), and engaging in a counting task (Boon et al., 2002). This effect was linearly related to the distraction provided by concomitant activities which affected the ability to correctly record the actual amount of food ingested (Higgs and Woodward, 2009; Marsh et al., 2015). Therefore, engaging in a task that diverts attention away from food could interfere with eating behavior and lead to greater caloric intake.

Similarly, studies using multimedia devices show that the distraction produced by watching a television program interferes with the amount of food consumed (Hetherington et al., 2006; Bellissimo et al., 2007; Patel et al., 2011; Marsh et al., 2013; Ogden et al., 2017). In particular, television content affects attention influencing eating behavior (Chapman et al., 2014), regardless of appetite level (Blass et al., 2006). In addition, Moray et al. (2007) found that the amounts of food ingested were less accurate if participants watched television while eating.

The distraction provided by smartphone use has also been highlighted in recent years (Kenney and Gortmaker, 2017). In a study conducted among 62 university volunteers between 18 and 28 years of both sexes, da Mata Gonçalves et al. (2019) evaluated the distraction produced by smartphone use during meals on both the total caloric intake and the type of calories ingested. During the trial sessions, participants were introduced to a snack task in which they were asked to eat under three different experimental conditions: no distraction, using their smartphones, or reading a printed text. After each session, the total calorie intake and nutritional composition of the food ingested were measured. The results showed that eating in the presence of distractors (smartphone/reading of printed articles) increased the total calorie intake by 15% with higher lipid ingestion. These results showed that smartphone use during meals, as well as reading a printed text, significantly affects the number of calories ingested.

Further, taking into account the external factors influencing eating behavior, coupled to the most recent observations concerning distraction effects on the number of calories ingested, Lopez et al. (2019a) tested the hypothesis by which children engaged in smartphone media multitasking activities (MMT) would be more driven by environmental stimuli. In their study, they verified the relationship between media multitasking—the use and switching from unrelated forms of digital media—and obesity risk. The authors recruited a sample of 179 pre-adolescent children aged 9–11 years and investigated the relationship between media multitasking and BMI. Their results showed a positive association between the frequency of children’s MMT behavior and BMI, regardless of physical activity, suggesting that the use of screen time technologies affects food intake by diverting attention to external stimuli. In line with previous findings, individuals eat more as a result of reduced cognitive control about the amount of food ingested (Ogden et al., 2013; Dohle et al., 2017).

Psychological research examined in more detail the role of attention during meals. Through experimental paradigms, it has been proved that eating distractedly increases both the current food intake and the amount of food consumed at subsequent meals (Higgs and Woodward, 2009; Higgs and Donohoe, 2011; Mittal et al., 2011; Oldham-Cooper et al., 2011; Ogden et al., 2017). Distractors significantly affect dietary memory formation, preventing the proper awareness of food ingested and interfering with hunger and satiety signals. Satiety is a key component of appetite control and refers to the feeling of fullness which suppresses additional intake (Blundell and Tremblay, 1995; Morris et al., 2020). It is the result of physiological processes that tend to split into early cognitive and sensory influences and, subsequently, into post-ingestive effects (Blundell and Tremblay, 1995; Bellisle and Blundell, 2013). More recent cognitive models of eating behavior suggested that satiety is partly cognitively constructed and memory dependent (Higgs et al., 2017). These models are supported by consistent evidence that reducing memory for food consumed by interfering with attention at the time of consumption increases subsequent food intake (Higgs and Woodward, 2009; Mittal et al., 2011; Oldham-Cooper et al., 2011; Robinson et al., 2013; Higgs, 2015). Besides, the impact of distraction on calorie intake could be related to the way that different types of dietary contexts influence an individual. According to Ogden et al. (2013), a possible explanation lies in the multidimensional nature of the distraction, which could affect the link between hunger and changes in the desire to eat. From this perspective, she proposed two forms of distraction: distraction away from hunger and distraction away from eating. Once external factors are distracted from internal stimuli, such as hunger and satiety, the individual eats mindlessly and food intake would not be encoded to affect their desire to eat. Nevertheless, food intake requires a certain cognitive effort in itself, and if too distracted, the subject will have insufficient cognitive resources to engage in eating behavior. To date, the influence of attention and memory on eating behavior is well known (Chieffi et al., 2011a,b, 2015; Robinson et al., 2014a; Higgs and Spetter, 2018), and neuropsychological evidence shows that memory deterioration, or amnesia, corresponds to increased food intake (Rozin et al., 1998; Higgs et al., 2008; Chieffi et al., 2017). Furthermore, experimental evidence shows that increased awareness of the calories ingested during previous meals reduced the number of calories ingested subsequently in both normal and overweight subjects (Higgs et al., 2008; Higgs and Donohoe, 2011; Robinson et al., 2014b; Seguias and Tapper, 2018). Similarly, weight reduction treatments, which limited the time spent watching television or playing video games during meals, produced a greater decrease in BMI (Robinson, 1999). However, no correlation between digital device use and caloric intake has also been reported (Whitelock et al., 2018; Whitelock and Robinson, 2018) and further investigations are needed.

## Smartphone and Social Facilitation

The main difference between smartphones and traditional digital technologies is that the phone provides an easier way to enjoy intrinsically social activities. Studies investigating mobile phone usage models reveal that social interaction and peer chat features are the most used, and teenagers are estimated to send over 110 messages per day (Lenhart, 2015; Smith and Page, 2015; Teo et al., 2018).

Smartphone use implies that adolescents engaging in multitasking activities during meals interact with friends and family in a distinctly different way than other screen time devices. It has been observed that individuals tend to overeat in the presence of friends and family, so social activity with their smartphones during meals could play a significant role in eating behavior.

The construction of “social facilitation” implies that people tend to change their behavior according to others’ behavior (Herman, 2015). Concerning eating behavior, eaters tend to activate food intake in people who do not eat (de Castro and de Castro, 1989; de Castro, 1997). In the same way, the presence of passive individuals who do not eat will make eaters more aware of their behavior, inducing them to decrease food consumption (Herman, 2015).

It has been shown that simple companionship can increase food intake by about 44% (de Castro and de Castro, 1989; de Castro, 1997), exerting a facilitating effect that manifests itself independently of real food needs (de Castro and de Castro, 1989). Moreover, this effect is cross-cultural (Herman, 2015).

Several studies indicated that social influence is so pervasive that even a simple online presence through digital technologies is enough to trigger facilitation effects in a variety of activities ranging from labyrinth resolution and arithmetic tasks to physical exercise (Park and Catrambone, 2007; Anderson-Hanley et al., 2011; Snyder et al., 2012).

Extending these results, Teo et al. (2018) tested the hypothesis by which the virtual presence of friends and parents—interconnected through a telephone messaging service—exerts a social facilitation effect on eating behavior. In a study of 50 Singaporean male adolescents, they examined whether social activity via smartphone could affect the number of calories ingested. Participants were randomly assigned to one of the following telephone activities: (i) sending and receiving messages (social activity) or (ii) reading a neutral article (non-social activity). Their results showed that participants consumed more calories when interacting virtually than those reading the article.

These findings seem to confirm the scientific literature highlighting the role of social influences on food intake (de Castro, 1997; Herman, 2015) and suggested that different ways of smartphone use may influence individuals to eat more than required. However, as suggested by the authors, virtual social facilitation is an emerging concept which needs further investigations.

Using mobile phones, people interacting via messaging service cannot be considered as eating co-actors. Analogously, people receiving messages are not aware of the amount of food their counterpart eats, minimizing the importance of maintaining an impression through food intake.

To date, virtual social facilitation cannot be described in the same way of a group exerting co-action or passive audience effects, and future research will have to investigate whether the impact of social facilitation can also apply to the digital realm.

## Discussion

In recent years, smartphone use has progressively increased in the youth population, especially during meals (Kabali et al., 2015).

As reported in our review, preliminary findings suggest that distraction and social facilitation can be taken into account to explain the link between smartphone use and food intake. However, although a social facilitation effect has been observed even in a virtual context, further investigations should exclude any alternative cognitive explanations (Teo et al., 2018). Indeed, participants engaged in a social interaction activity via smartphone messaging service could eat more because they were more distracted (Bellisle et al., 2004; Brunstrom and Mitchell, 2006; Robinson et al., 2013) rather than because the act of messaging was social.

As reported in the above sections, smartphone use affects food intake by diverting attention from eating behavior; with reduced cognitive resources, the user engages in “mindless eating” and consumes more food (Ogden et al., 2013; Dohle et al., 2017).

Some potential mechanisms have been proposed to explain the role of distraction in eating behavior (La Marra et al., 2009; Robinson et al., 2014a; Higgs, 2015; Higgs and Spetter, 2018). However, more recently, it has been highlighted that the perceptual load theory could be successfully applied to the study of ingestive behavior (Morris et al., 2020). The perceptual load theory is a key theory in the literature on selective attention and implies that the extent to which task-irrelevant stimuli are processed is regulated by attention availability (Lavie, 2005, 2010). It is a passive process carried out automatically by the perceptual system at an early stage of selection and is determined by whether the primary task leaves adequate spare perceptual capacity (Lavie, 2005, 2010). Similarly, it has been suggested that appetite control based on satiety could be altered when attention is absorbed in a perceptually demanding task (Morris et al., 2020). A reliable satiety response is provided by cognitive and physiological stimuli integration (Yeomans and Chambers, 2011; Chambers et al., 2013; Camps et al., 2016; Chen et al., 2016; McCrickerd et al., 2020). Therefore, physiological signals can be altered by the perceptual load, affecting appetite control during consumption. These results are based on existing models of appetite regulation (Bellisle and Blundell, 2013), which emphasize the role of cognitive influences on satiety.

Although the role of biological, environmental, and cultural factors in determining dietary behavior is widely recognized (Monda et al., 2017; Qasim et al., 2018), recent experimental evidence also supports the role of cognition in satiety (Higgs et al., 2017), which could be altered by a high perceptual load. Therefore, factors acting on satiety (such as post-ingestive stimuli derived from nutrients) may also depend on the availability of basic perceptual capacity. The perceptual load is known to substantially interfere with the processing of information, from the early stages of perceptual elaboration to the encoding of memory, as indexed by both behavioral and neural measurements (Lavie, 2005, 2010).

A further interpretation points to the role of dopaminergic pathways. These are mainly implied in the “reward dependence” mechanisms. These mechanisms are related to the activity of projections to the limbic areas, mainly exerting facilitation, and controlled by the prefrontal cortex which inhibits (DLPFC) or stops (orbitofrontal cortex) food assumption, in particular as regards its compulsive-like behaviors (Lopez et al., 2019b). On the other hand, the same areas are heavily implied in attentional processes (Supervisory Attentional System—SAS; Shallice et al., 1989), in the cognitive estimation of stimuli of the environment (including the food) and the consequences of eating behaviors. It is possible that the allocation of resources to stimuli other than food (the smartphone) could divert the frontal areas from exerting executive control on food assumption.

Furthermore, another neurobiological mechanism could be ascribed to the role of serotonergic pathways. These are mainly related to “harm avoidance” behaviors. According to the theory of serotonin/dopamine balance (Cools et al., 2011), increased activity of dopaminergic pathways entails reduced activity of serotonergic ones. Attempting to include all these insights in a coherent pathophysiological framework, we would suggest the following cascade of events: (1) The reward dependence activates dopaminergic extrafrontal pathways (in particular mesolimbic); (2) the interfering stimuli prevent frontal areas to exert normal control on the cognitive estimation of food assumption and/or to stop the calorie intake; and (3) the imbalance of dopaminergic/serotonergic mechanisms led to acting worrying food-related behaviors. Finally, the well-known neurobiological mechanisms of long-term potentiation and neural plasticity may give rise to a stable pattern of eating behaviors and to increase the risk of affective disorders like depression.

This evidence seems to be particularly worrying considering that mobile phone overuse in some cases represents a risk behavior comparable to addiction (Domoff et al., 2020).

Our review underlines that the use of mobile devices during meals interferes with eating behavior contributing to calorie increase in a segment of the population for which the international scientific community is particularly concerned. This knowledge could help to inform cognitive dietary interventions about the importance of encouraging participants to pay attention to food intake.

## Author Contributions

All authors read and approved the final manuscript.

## Conflict of Interest

The authors declare that the research was conducted in the absence of any commercial or financial relationships that could be construed as a potential conflict of interest.
